# Supplementary material for: Explainable death toll motion modeling: COVID-19 data-driven narratives
Source: PLoS One. 2022 Apr 8;17(4):e0264893. doi: 10.1371/journal.pone.0264893 (PMC8993006; doi:10.1371/journal.pone.0264893)
Supplement: S1 Appendix — However, the complete list of considered factors is much larger. Tables 7–12 show the factors not discussed previously. The names we gave to these factors are self-explanatory. (PDF) [file pone.0264893.s001.pdf]

S1 Appendix. Tables 1, 2, 3, and 4 present the main factors found by Algorithm 1. However, the complete list of considered factors is much larger. Tables 7, 8, 9, 10, 11, and 12 show the factors not discussed previously. The names we gave to these factors are self-explanatory.

Table 7. Health system.

Out-of-pocket health expenditure (% health expenditure)
Gov. health expenditure (% health expenditure)
Gov. health expenditure (% of general expenditure)
Hospital beds needed
Critical care beds needed
Invasive ventilators needed
Tests performed
Hospital beds needed (per 100k)
Critical care beds needed (per 100k)
Invasive ventilators needed (per 100k)
Tests performed (per 100k)
Hospital bed normal usage
Critical care bed normal usage
Hospital beds available
Critical care beds available
Hospital beds needed/available
Hospital beds available (per 100k)
Critical care beds available (per 100k)
Doctors available (per 100k)
Nurses available (per 100k)

Table 8. Derivative information.

Hospital beds needed on previous day
Critical care beds needed on previous day
Invasive ventilators needed on previous day
Tests performed on previous day
Hospital beds needed on previous day (per 100k)
Hospital beds needed on previous day (per 100k)
Invasive ventilators needed on previous day (per 100k)
Tests performed on previous day (per 100k)
Hospital beds available on previous day
Critical care beds available on previous day
Hospital beds needed/available on previous day
Critical care beds needed/available on previous day

Table 9. Causes of death.

Deaths from Iron deficiency
Deaths from Zinc deficiency
Deaths from Vitamin-A deficiency
Deaths from diet high in sodium
Deaths from diet low in calcium
Deaths from cancer, 15-49 years
Deaths from cardiovascular diseases, 15-49 years
Deaths from diabetes, 15-49 years
Deaths from cancer, 50-69 years
Deaths from cardiovascular diseases, 50-69 years
Deaths from cancer, 70+ years
Deaths from cardiovascular diseases, 70+ years
Deaths from diabetes 70+ years

Table 10. Vaccination.

BCG vaccination started (years)
BCG vaccination stopped (years)
BCG coverage (%)

Table 11. Population concentration.

Urban areas over 500k inhabitants
Urban areas over 1M inhabitants
Urban areas over 2M inhabitants
Urban areas over 5M inhabitants
Agglomerations of 500k inhabitants (% of urban population)
Agglomerations of 1M inhabitants (% of urban population)
Agglomerations of 2M inhabitants (% of urban population)
Agglomerations of 5M inhabitants (% of urban population)

Table 12. Population.

Age working dependency ratio
Population
